# Insect derived extra oral GH32 plays a role in susceptibility of wheat to Hessian fly

**DOI:** 10.1038/s41598-021-81481-4

**Published:** 2021-01-22

**Authors:** Subhashree Subramanyam, Jill A. Nemacheck, Victor Bernal-Crespo, Nagesh Sardesai

**Affiliations:** 1 Crop Production and Pest Control Research Unit, USDA-ARS, West Lafayette, IN USA; 2 Department of Entomology, Purdue University, West Lafayette, IN USA; 3 College of Veterinary Medicine, Purdue University, West Lafayette, IN USA; 4 Corteva Agriscience, Johnston, IA USA

**Keywords:** Molecular biology, Plant sciences

## Abstract

The Hessian fly is an obligate parasite of wheat causing significant economic damage, and triggers either a resistant or susceptible reaction. However, the molecular mechanisms of susceptibility leading to the establishment of the larvae are unknown. Larval survival on the plant requires the establishment of a steady source of readily available nutrition. Unlike other insect pests, the Hessian fly larvae have minute mandibles and cannot derive their nutrition by chewing tissue or sucking phloem sap. Here, we show that the virulent larvae produce the glycoside hydrolase MdesGH32 extra-orally, that localizes within the leaf tissue being fed upon. MdesGH32 has strong inulinase and invertase activity aiding in the breakdown of the plant cell wall inulin polymer into monomers and converting sucrose, the primary transport sugar in plants, to glucose and fructose, resulting in the formation of a nutrient-rich tissue. Our finding elucidates the molecular mechanism of nutrient sink formation and establishment of susceptibility.

## Introduction

Significant global economic damage is caused by the infestation of maize, wheat and rice by insect pests, resulting in annual production losses ranging from around 45–75 metric megatons^1^. While there are several factors that play crucial roles in defense, the plant cell wall, composed of a heterogeneous mixture of high-molecular weight complex polysaccharides such as cellulose, hemicellulose, pectin, and diverse proteins, in addition to providing structural support, is the primary line of defense against pests^2^. Therefore, it is imperative for virulent pests to disrupt the intricate cell wall barrier and effectively deliver effector proteins, enzymes, and/or toxins. Once plant tissue is ingested, to breakdown the complex wall polysaccharides and release nutrients, phytophagous insects produce an array of plant cell wall degrading enzymes (PCWDEs) that target cell wall components and have major effects on wall architecture^3–5^. PCWDEs are a subset of Carbohydrate-Active enZymes (CAZy) that exist in large multigene families, encode diverse proteins, and exhibit varied expression patterns and functional specialization across diverse taxonomic groups^6^.

In insects, PCWDEs primarily include enzyme families such as glycoside hydrolases, polysaccharide lyases, and carbohydrate esterases^7^. Glycoside hydrolases (GHs) constitute one of the largest families (GH1-GH167) of PCWDEs, as catalogued in the CAZy database (http://www.cazy.org). GHs have been cloned and characterized from several insect species belonging to different orders that contribute to cell wall digestion and deconstruction^3–5,7–9^. Novel PCWDEs belonging to the GH32 family, encoding β-fructofuranosidase or invertase, have been characterized from sugarcane weevil, *Sphenophorus levis*^8^, and emerald ash borer, *Agrilus planipennis*^9^. Increasing evidence, via next-generation sequencing technologies and phylogenetic studies, reveals horizontal gene transfer (HGT) of GHs from prokaryotes to insects over the course of evolution^10^.

The Hessian fly (*Mayetiola destructor* [Say]), a dipteran gall midge (family: Cecidomyiidae), is an obligate pest on host wheat (*Triticum aestivum*) that causes severe monetary losses^11^. The interaction of wheat with Hessian fly involves gene-for-gene interactions^12^. During compatible interactions, within three days after egg hatch (DAH), the larvae alter host metabolic pathways upregulating genes associated with susceptibility^13–15^ resulting in the formation of sugar and protein-rich nutritive tissue^16^ that provides a nutrient-rich diet^15,17,18^ allowing the larvae to complete their life cycle thereby making the plant susceptible and stunted^19^. In contrast, the incompatible *H* (Hessian fly resistance) gene-mediated resistance is accompanied by increased accumulation of defense antinutrient proteins^20–23^ that disrupt the insect midgut microvilli^24^ resulting in larval death within 4–5 DAH, and the plant shows normal growth. It is believed that to complete their life cycle and development, the larvae must be able to inject salivary effector proteins^25^, induce permeability in the plant^26–28^, suppress plant defense responses^13,29^, alter host plant physiology^17,30^ and facilitate the diffusion of nutrients to the feeding site^16^. Consistent with induced susceptibility^16^ the plant cells are converted into a nutrient sink, nourishing the developing virulent larvae.

Here, we report not only the expression of *MdesGH32*, a gene belonging to the GH32 family in Hessian fly larvae feeding on susceptible wheat, but also presence of the larval protein in the feeding sites on the host plant. The homolog of this gene in the Great Plains (GP) biotype of Hessian fly is one of a group of ten *Mayetiola destructor levanase* (*MDL*) genes identified recently to belong to the GH32 family^31^. With the MDL proteins being demonstrated to have levanase/inulinase/sucrase activities, and present in digestive tissues within the Hessian fly larvae^31^, our results complement and extend the previous work to strongly support the possible involvement of extra-oral insect PCWDEs in breakdown of wheat cell wall architecture and carbohydrate components by Hessian fly, and elucidate the molecular mechanism by which a nutrient sink is established. Further molecular and functional characterization of this PCWDE could reveal possible virulence mechanisms employed by Hessian fly during compatible plant–insect interactions and lead to further downstream applications for efficient management and control of this and other devastating dipteran insect pests.

## Results

### MdesGH32 secondary structure is similar to an invertase

**Figure 1:**
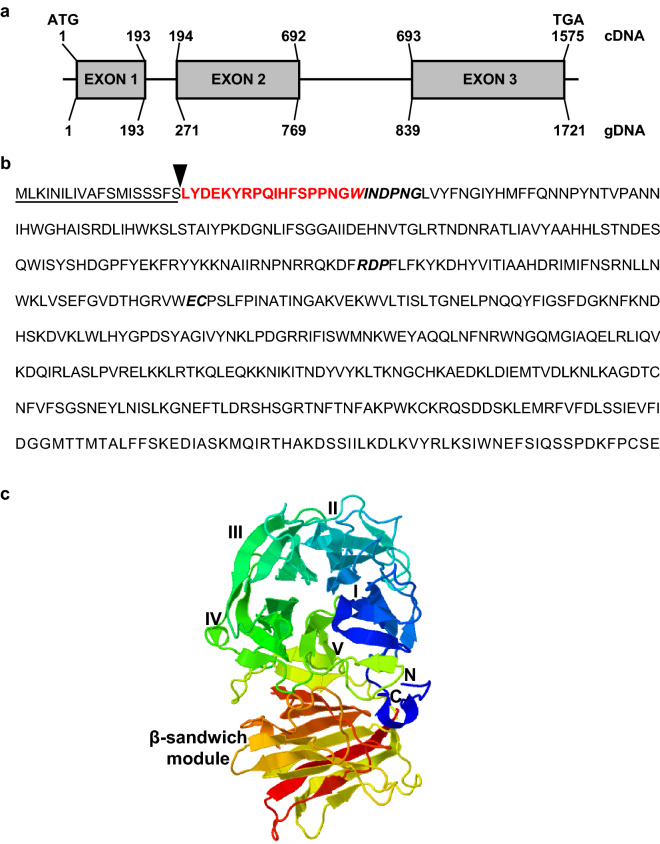
MdesGH32 sequence characterization. (**a**) Schematic representation of coding (cDNA) and genomic (gDNA) sequences of *MdesGH32*. The exons are represented by rectangular boxes. (**b**) Protein sequence of MdesGH32 showing three conserved motifs (bold and italicized font) of glycoside hydrolase 32 proteins. Underlined amino acids represent the putative signal peptide with the cleavage site marked by a filled triangle. Amino acids in red, bold font depict the sequence used for peptide antibody synthesis. (**c**) Modeled secondary structure of MdesGH32 showing presence of a five-bladed β-propeller (I, II, III, IV, V) structure at the N-terminus (N) that is connected to a β-sandwich module at the C-terminus (C) resembling the structure of invertase.

The open reading frame (ORF) of *MdesGH32* cloned from Biotype L Hessian fly was 1575-bp long (GenBank accession no. MT179729), encoding a predicted polypeptide of 524 amino acids (GenBank accession no. QIZ03396), with a predicted molecular mass of 60.9 kDa and a pI of 9.29. The genomic sequence of *MdesGH32* was 1721-bp long comprising of two introns of lengths 77 and 69 bp, starting at positions 194 and 770, respectively (Fig. 1a). Sequence alignment of the Biotype L *MdesGH32* and JBrowse annotated sequence from the draft Biotype GP genome^32^ revealed 98.5% and 97.9% nucleotide and protein sequence identity, respectively, identified as *MDL-9*^31^. A putative secretory signal peptide sequence of 19 amino acids at the N-terminus was predicted by SignalP (Fig. 1b). Analysis of the MdesGH32 amino acid sequence revealed conserved domains for the glycoside hydrolase family GH32 between positions 30–340 (N-terminal domain) and 343–502 (C-terminal domain). The secondary structure model of the MdesGH32 protein sequence (Fig. 1c) was based on the template C3KF5A, an invertase from *Schwanniomyces occidentalis*^33^, with which 29% of identity is shared. 90% (473 residues) of the MdesGH32 sequence was modeled with 100% confidence using this template. Resembling other GH32 family proteins, MdesGH32 possesses a five-bladed β-propeller N-terminal catalytic domain, each having four antiparallel β-strands with a characteristic ‘W’ arrangement around the central axis (Fig. 1c). These β-sheets enclose the cavity of the active site that has three conserved amino acids (D40, D171, and E218)^34,35^. The conserved amino acids of the ‘catalytic triad’, located in the active site are part of the WINDPNG (β-fructosidase motif), RDP and EC motifs (Fig. 1b) and are indispensable for substrate binding and catalysis^35,36^. MdesGH32 is also characterized by two 6-stranded β-sheets consisting of antiparallel β-strands at the C-terminus of the catalytic domain, a distinct trait of GH32 enzymes, creating a sandwich-like fold (Fig. 1c).

### MdesGH32 in Hessian fly originated due to horizontal gene transfer

GH32 sequences from various sources including plants, insects, bacteria and fungi were used to construct a maximum-likelihood phylogenetic tree. The analysis revealed clustering of GH32 sequences into four distinct clades (Supplementary Fig. 1). Interestingly, *MdesGH32* clustered in clade III with predominance of bacterial GH32 sequences and very few insect GH32 sequences. The insect sequences included those from *Contarinia nasturtii* (swede midge; Order: Diptera), *Leptotrombidium deliense* (mite; Order: Trombidiformes) and *Spodoptera litura* (tobacco cutworm; Order: Lepidoptera). MdesGH32 was distantly related to GH32 sequences from other insects, fungi and plants that grouped together in their respective clades (Supplementary Fig. 1).

### Expression of MdesGH32 increases during larval virulence

**Figure 2:**
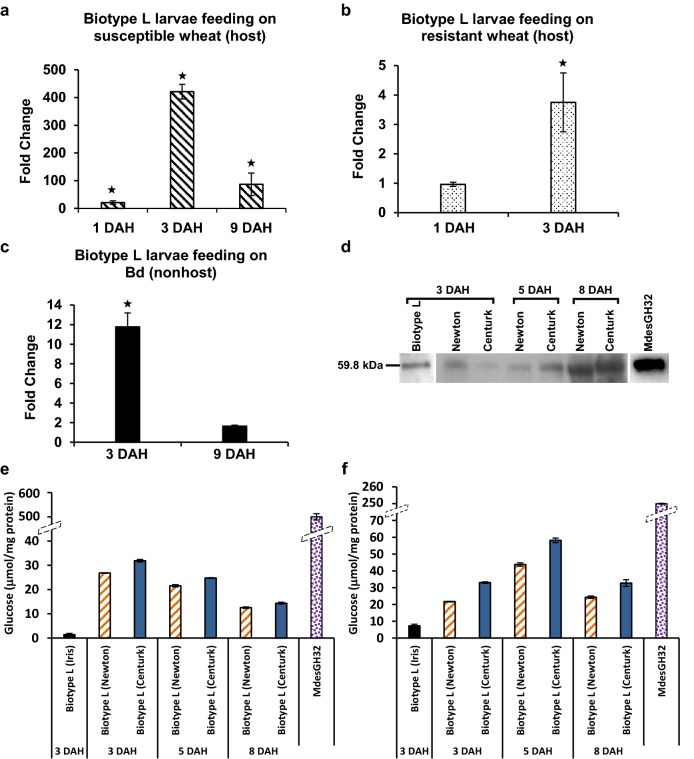
Temporal expression and activity of *MdesGH32*. Transcript levels of *MdesGH32* in (**a**) Virulent Biotype L Hessian fly larvae feeding on Newton (susceptible) host wheat. (**b**) Avirulent Biotype L Hessian fly larvae feeding on Iris (resistant) host wheat. (**c**) Biotype L Hessian fly larvae feeding on nonhost *Brachypodium distachyon*. Values are the fold change ± SE for transcript levels as compared to neonate larvae (collected on the day of egg hatch). Statistically significant (*p* < 0.05) differences are indicated by “*”. Error bars represent data from three biological replicates (*n* = 3; each with three technical replicates). (**d**) Immunodetection of MdesGH32 in susceptible wheat plants 3, 5 and 8 DAH, and virulent Biotype L larvae 3 DAH (first lane; run on a different gel). Purified recombinant MdesGH32 protein was used as a positive control (last lane; run on a different gel) and showed a single band of the expected size of 59.8 kDa. (**e**) Invertase activity of MdesGH32. (**f**) Inulinase activity of MdesGH32. Enzyme activities are quantified as μmol glucose/mg protein in Biotype L larvae from resistant (Iris), and susceptible (Newton and Centurk) wheat lines, and recombinant MdesGH32 and are represented as the mean ± SE from three biological replicates (*n* = 3) for each sample.

*MdesGH32* transcripts accumulated to higher levels at all time points (1, 3 and 9 DAH) in virulent larvae of Biotype L feeding on susceptible Newton wheat plants as compared to neonates (Fig. 2). While the transcripts increased to 21-fold (*p* < 0.001) by 1 DAH, peak expression was observed by 3 DAH with a sharp increase of 421-fold (*p* = 0.0007) followed by a decline to 87-fold (*p* < 0.0001) by 9 DAH (Fig. 2a). Increased *MdesGH32* transcripts were also observed in avirulent larvae feeding on resistant Iris wheat, however, the levels were significantly lower than those observed in the virulent larvae. The transcripts accumulated to only 3.75-fold (*p* = 0.005) by 3 DAH and were not significantly different at 1 DAH as compared to the neonates (Fig. 2b). The expression profile of *MdesGH32* in Biotype L larvae feeding on nonhost *Brachypodium distachyon* (Bd) plants also showed increased transcript abundance (Fig. 2c). At 3 DAH the transcripts increased moderately by 11.8-fold (*p* = 0.0002), but showed no significant change in expression by 9 DAH (*p* = 0.3841) as compared to the neonates (Fig. 2c).

### MdesGH32 is secreted extra-orally into susceptible wheat

MdesGH32 (lacking the signal peptide sequence) was expressed in *E. coli* BL21 (DE3) cells and affinity-purified using Ni–NTA chromatography. The purified MdesGH32 was homogeneous, yielding a single band with a molecular mass of 59.8 kDa as detected using anti-MdesGH32 antibody by gel blot analysis (Fig. 2d and Supplementary Fig. 2). MdesGH32 was also observed in the larval protein extracted from virulent Biotype L larvae feeding on Newton wheat (Fig. 2d and Supplementary Fig. 2). As the peptide sequence used to produce the polyclonal antibody was 66.7–83.3% identical to other MDL gene homologs, the possibility of the antibody cross-reacting with other MDL proteins, in addition to detecting MdesGH32, cannot be excluded. To determine whether MdesGH32 from the larvae was secreted on the plant, we carried out gel blot analysis with total plant protein extracted from the larval feeding sites in two susceptible wheat lines at 3, 5 and 8 DAH. To prevent any protein contamination from larvae, all the larvae were removed from the crown tissue during collections from these susceptible wheat lines. Gel blot analysis revealed the presence of MdesGH32 in the total plant proteins prepared from both susceptible wheat lines (Fig. 2d and Supplementary Fig. 2). The band intensity in the susceptible wheat lines was faint at 3 and 5 DAH but increased strongly by 8 DAH (Fig. 2d). Gel blot analysis of the purified recombinant MdesGH32 with the pre-immune serum did not yield any positive signal (Supplementary Fig. 3a).

### MdesGH32 exhibits invertase and inulinase activity

To functionally characterize MdesGH32, the purified recombinant protein was tested for enzyme activity using different substrates. MdesGH32 was able to hydrolyze sucrose and inulin substrates thus confirming that it has both invertase (Fig. 2e) and inulinase (Fig. 2f) activities. MdesGH32 exhibited higher invertase (Fig. 2e) specific activity as compared to that of inulinase (Fig. 2f). MdesGH32 did not show levanase activity (data not shown). We also assessed invertase and inulinase activities in total larval protein extracted from first-instar avirulent (3 DAH) larvae feeding on resistant Iris wheat and both first- (3 and 5 DAH) and second-instar (8 DAH) virulent larvae feeding on two susceptible wheat lines. In general, as compared to the avirulent larvae, the virulent larvae showed higher levels of both invertase (Fig. 2e) and inulinase (Fig. 2f) activities. In the virulent larvae the highest specific activity of invertase was observed on 3 DAH (Fig. 2e), while highest inulinase activity was observed by 5 DAH (Fig. 2f).

### MdesGH32 is localized inside the plant tissue

**Figure 3:**
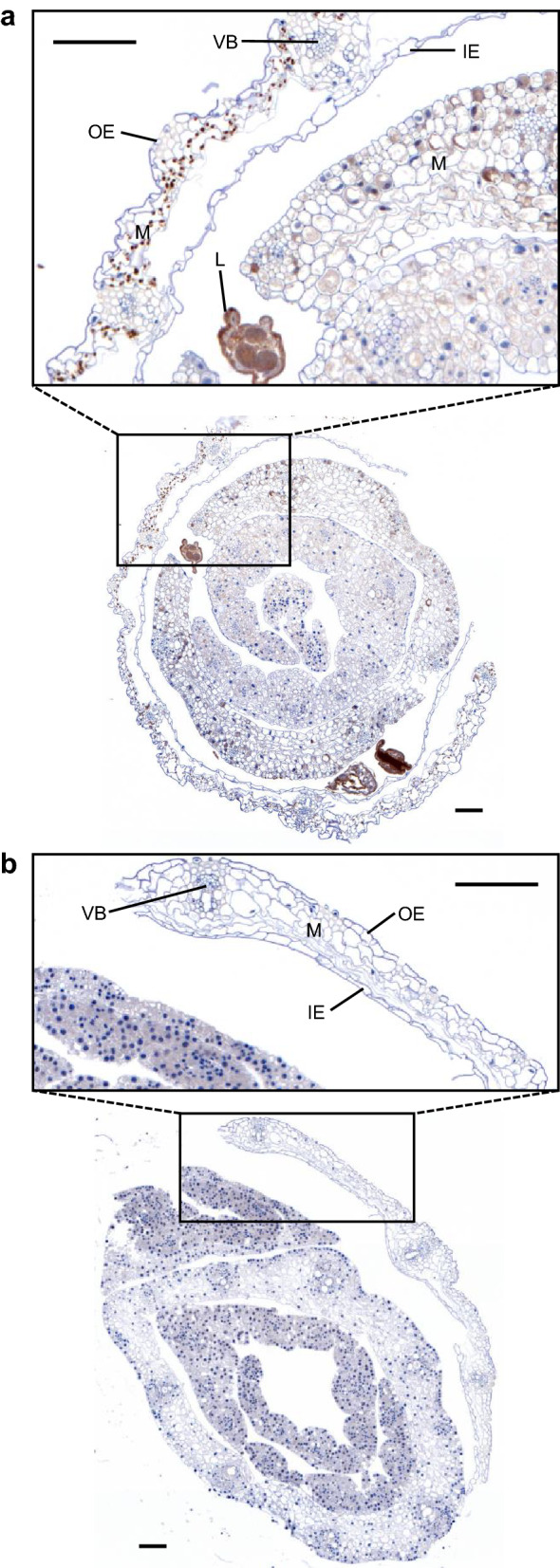
Immunohistochemical localization of MdesGH32 in wheat tissues. (**a**) Biotype L-infested Newton crown tissue collected 7 DAH showing MdesGH32 localized (brown spots) throughout the mesophyll cells (M) between the outer epidermis (OE) and inner epidermis (IE) of the leaf sheath (inset) being fed on by larvae (L). (**b**) Uninfested Newton tissue showing intact mesophyll cells with no MdesGH32 staining (inset). *VB* vascular bundle; scale bar, 100 µm.

To determine whether the extra-oral MdesGH32 is surface-localized or present within the wheat plant tissue we carried out immunohistochemical localization studies in Biotype L-infested Newton wheat plants (Fig. 3). Distinct brown chromogens resulting from the DAB staining were seen in the mesophyll cells as well as the outer and inner epidermis of leaf sheaths being fed upon by the Biotype L larvae in infested Newton crown transverse sections, thus confirming the presence of MdesGH32 within the susceptible plant tissue (Fig. 3a). In contrast, the uninfested control Newton crown tissue conspicuously lacked the brown chromogens, indicating the high specificity of the DAB staining method in this system (Fig. 3b). The Biotype L-infested Newton tissue did not show the presence of the brown chromogens when probed with the pre-immune serum (Supplementary Fig. 3b).

### Cell wall permeability increases in susceptible wheat

**Figure 4:**
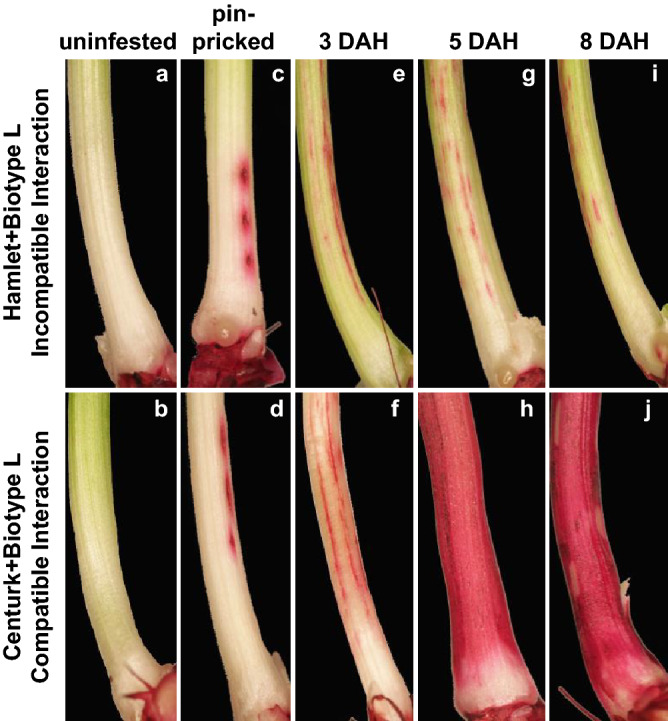
Temporal changes in plant cell wall permeability. Resistant (Hamlet) and susceptible (Centurk) wheat plants stained with neutral red (NR) to assess cell wall permeability at 3 (**e**,**f**), 5 (**g**,**h**) and 8 (**i**,**j**) DAH. Uninfested plants from both wheat lines were stained with NR as negative controls (**a**,**b**). Some plants from both wheat lines were also pin-pricked (**c**,**d**) and stained to distinguish staining caused by larval feeding from that caused by physical damage during dissections. Top panels show NR stained Hamlet plants while bottom panels show NR stained Centurk plants. The resistant plants showed limited distribution of NR stain appearing as blush, spots, broken lines and solid lines. The susceptible plants showed intense staining as dark streaks and lines covering the entire length of the crown tissue.

Since the susceptible wheat line showed presence of MdesGH32 protein that has the potential to breakdown cell wall polymers, we assessed the cell wall permeability levels in resistant (Hamlet) and susceptible (Centurk) plants by Neutral Red (NR) staining at 3, 5 and 8 DAH (Fig. 4). None of the uninfested plants for either of the wheat lines were stained (Fig. 4a,b) unless wounded by a pin (Fig. 4c,d). Permeability was observed in both the resistant and susceptible wheat lines at 3 (Fig. 4e,f), 5 (Fig. 4g,h) and 8 (Fig. 4i,j) DAH time points. However, in the resistant wheat lines the staining appeared as a smear, blush or solid lines (Fig. 4e,g,i), restricted at the larval feeding site (base of the crown tissue) with an average NR score remaining the same, temporally (3.1, 3.3, and 3.2 on 3, 5 and 8 DAH, respectively; Supplementary Table 1). In contrast, the susceptible wheat line showed intense NR staining with dramatic increase from 3 to 8 DAH (Fig. 4f,h,j). The NR scores were much higher in the susceptible wheat line as compared to those observed in the resistant wheat lines and increased gradually from 4.4 by 3 DAH to 6.5 by 8 DAH (Supplementary Table 1).

### Glucose levels increase in Hessian fly-infested susceptible wheat

**Figure 5:**
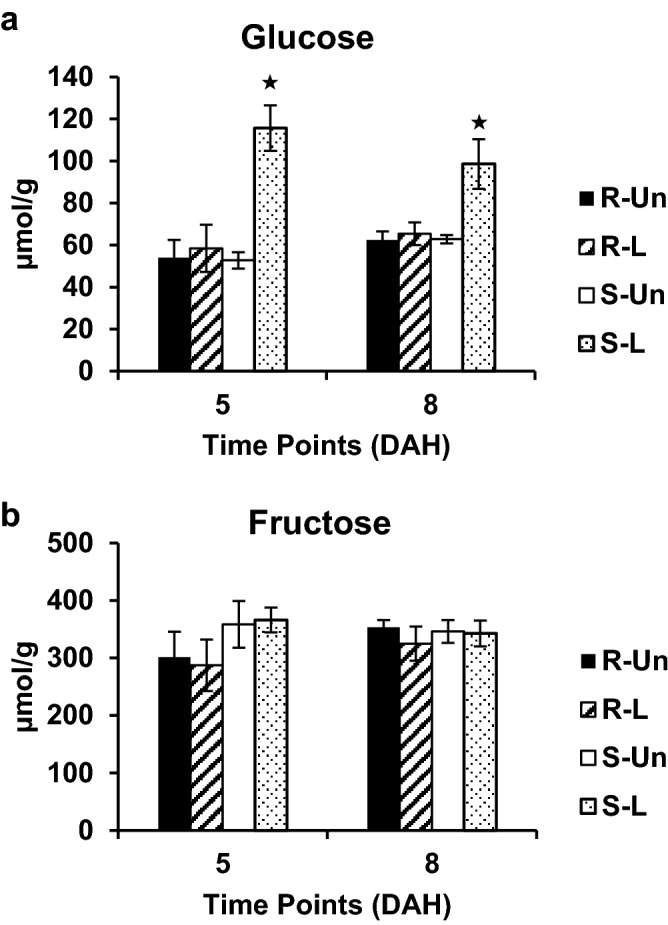
Wheat monosaccharide levels. (**a**) Glucose levels. (**b**) Fructose levels. Levels of monosaccharides, glucose and fructose measured as μmol/g of tissue in Hessian fly-infested resistant Hamlet and susceptible Centurk wheat crown tissue at 5 and 8 DAH. Black solid bar (R-Un) indicates uninfested resistant wheat plants; diagonal bar (R-L) indicates resistant wheat plants infested with Biotype L, white bar (S-Un) indicates uninfested susceptible wheat plants; dotted bar (S–L) indicates susceptible wheat plants infested with Biotype L. Error bars represent mean ± SE of three independent biological replicates (*n* = 3). Statistically significant (*p* < 0.05) differences in monosaccharide levels between infested and uninfested (control) wheat plants are indicated by “*”.

To determine if the extra-oral invertase and/or inulinase activity of MdesGH32 contributes in the breakdown of plant sucrose polymers into glucose and fructose monomers, UPLC-MS analysis was performed in resistant and susceptible wheat seedlings following Hessian fly infestation (Fig. 5). Results revealed significantly higher level of glucose by 5 (2.2-fold; *p* < 0.0001) and 8 (1.6-fold; *p* < 0.01) DAH in susceptible wheat seedlings compared to uninfested plants (Fig. 5a). There was no significant change in glucose levels in resistant wheat seedlings at any of the time points compared to the controls (Fig. 5a). Interestingly, unlike glucose, fructose levels did not change in either the resistant or susceptible wheat seedlings as compared to their uninfested controls (Fig. 5b).

### Expression of *TaFRK* increases in susceptible wheat

**Figure 6:**
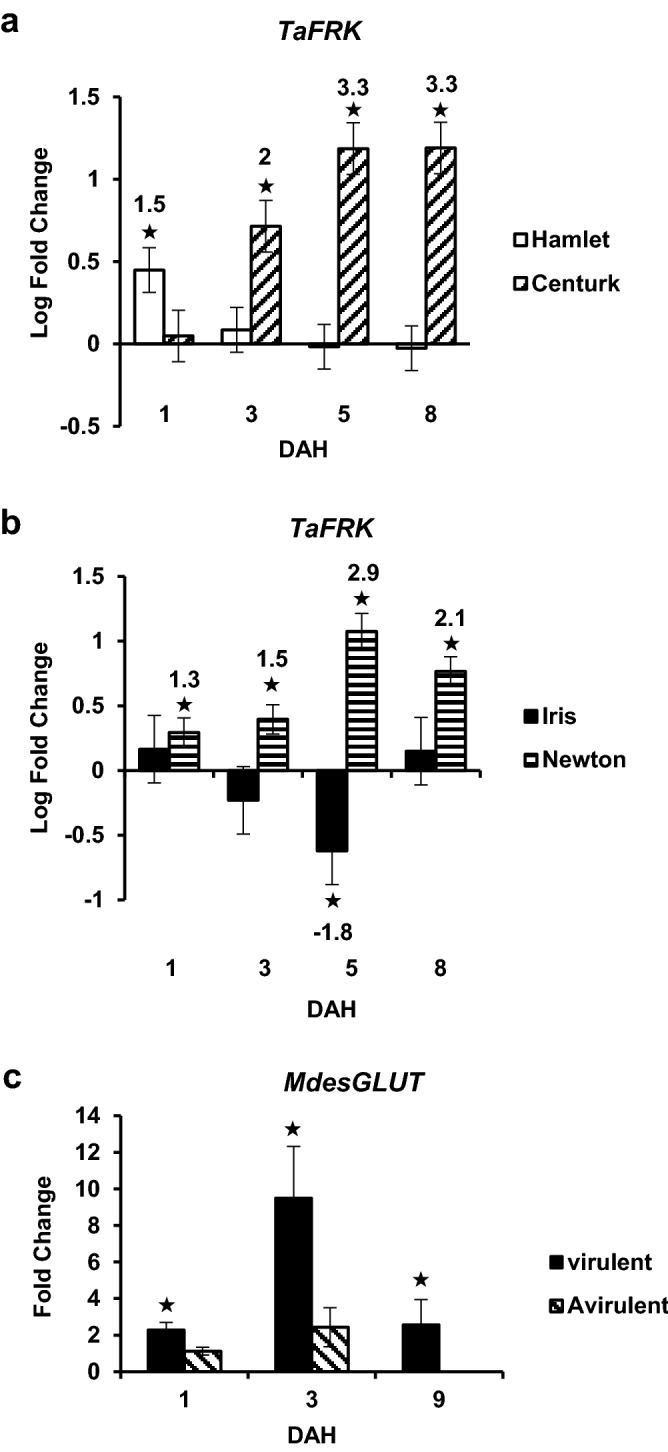
Expression of monosaccharide metabolism/transport genes in wheat and Hessian fly larvae. Transcript levels of *TaFRK* in (**a**) Hamlet (resistant, white bars); Centurk (susceptible; diagonal bars) and (**b**) Iris (resistant, black bars); Newton (susceptible; parallel bars) wheat lines at 1, 3, 5 and 8 DAH. Values are the logarithmic fold change ± SE of transcript levels in infested wheat samples as compared to the uninfested control at respective time points. Significant fold change values are shown on top of each bar. (**c**), Transcript levels of *MdesGLUT* in virulent (1, 3 and 9 DAH) and avirulent (1 and 3 DAH) Biotype L Hessian fly larvae feeding on susceptible (Newton) and resistant (Iris) wheat plants, respectively. Values are the fold change ± SE of transcript levels in virulent (black bars) and avirulent (diagonal bars) larvae as compared to neonate larvae (collected on the day of egg hatch). Error bars in all graphs represent data from three biological replicates (*n* = 3; each with three technical replicates). Statistically significant (*p* < 0.05) differences are indicated by “*”.

To determine whether fructose, formed from the hydrolysis of inulin and sucrose is phosphorylated for entering metabolic pathways, transcript levels of *TaFRK*, a fructokinase, were assessed. Significant *TaFRK* transcript accumulation was observed in the susceptible wheat Centurk at 3, 5 and 8 DAH ranging from 2- to 3.3-fold (*p* < 0.001) as compared to uninfested control plants (Fig. 6a). On the other hand, the resistant line Hamlet only showed a 1.5-fold (*p* = 0.004) increase in *TaFRK* transcripts at 1 DAH, but no significant difference at other time points (Fig. 6a). Resembling Centurk, the other susceptible wheat line Newton also showed significant accumulation of *TaFRK* mRNA at all time points with a peak expression of 2.9-fold by 5 DAH (*p* < 0.0001) compared to the uninfested plants (Fig. 6b). The expression of *TaFRK* in the resistant line Iris was not significantly different at 1, 3 and 8 DAH, but was significantly down-regulated (1.8-fold; *p* = 0.03) at 5 DAH (Fig. 6b).

### *MdesGLUT* expression is elevated in virulent larvae

To determine whether increased glucose monomers in the susceptible plants as well as endogenous glucose monomers in the Hessian fly larvae are transported across the larval midgut epithelial layer, transcripts of *MdesGLUT*, a glucose transporter, were quantified in the Hessian fly larvae. Higher *MdesGLUT* transcript levels were observed at all times in both the first- and second-instar virulent Biotype L larvae compared to neonates. As early as 1 DAH, transcripts increased by threefold (*p* = 0.0158) and then by 3 DAH elevated to 9.5-fold (*p* < 0.001) (Fig. 6c). The transcript levels decreased to 2.5-fold (*p* = 0.015) in second-instar larvae (9 DAH) with respect to the neonates (Fig. 6c). Compared to the neonates, in the avirulent larvae, *MdesGLUT* transcript levels did not change significantly at any of the time points (Fig. 6c).

### Cell wall-related genes are suppressed in Hessian fly-infested susceptible wheat

To understand how virulence of Hessian fly on wheat plants may affect the cell wall at the feeding sites, temporal changes in the expression of 29 genes involved in the biosynthesis of cell wall components (cellulose, hemicellulose, pectin) and cell wall-associated proteins in two resistant and two susceptible wheat lines were determined by qRT-PCR (Supplementary Fig. 4). Most genes that were differentially expressed were significantly down-regulated in both the susceptible wheat lines Centurk and Newton at most of the time points as compared to their respective uninfested control plants. While some of these differentially expressed genes were also down-regulated in both the resistant wheat lines Hamlet and Iris at some of the time points, the expression was not significantly different as compared to the uninfested controls for most genes or time points. Resistant plants did show increased transcript accumulation of some genes at select time points in response to larval attack (Supplementary Fig. 4).

## Discussion

The efficient utilization of wheat host nutrients, following induced susceptibility, is key to successful development of Hessian fly larvae. Several studies show the successful manipulation and reprogramming of host plant physiology by Hessian fly larval feeding^15,17,30,37,38^. Another crucial factor in insect development and survival is the breakdown of the intricate dynamic plant cell wall network, that acts as the first line of defense against phytophagous insects, by enzymes such as polygalacturonases, amylases, lipases, peptidases and proteases, endogenously^5,39^ as well as extra-orally^40^, and subsequent effective digestion in the insect midgut.

The multigene family encoding GH32 proteins constitutes one such class of PCWDEs that have been cloned and characterized from various insects. GH32s from insects have diverse enzyme activities (CAZy database). These enzymes are expressed during various stages of insect development and play a crucial role in the digestion and metabolism of ingested plant-derived cell wall materials within the gut^8,31,41^. *MdesGH32*, identified from one of the most virulent Hessian fly Biotype L larvae, encodes for a GH32-family protein. The predicted secondary structure of MdesGH32 shares a similar core topology to most of the GH32s^42^ consisting of two domains, a catalytic N-terminal domain with a 5-bladed β-propeller architecture coupled to a C-terminal β-sandwich-like domain. This β-sandwich module is necessary for overall stability of the protein^43^ while the β-propeller contains the active site^,^^34,35^. Phylogenetic analysis of MdesGH32 places the protein in the clade consisting predominantly of GH32s of bacterial origin, along with three other insects, and distant from the Insecta GH32 clade. Of ten putative GH32 proteins (MDL1-10) identified recently from the Hessian fly biotype GP^41^, MdesGH32 shares a 98% identity with MDL-9. A recent study on the similarity and proximity of the multiple *MDL* genes on a single scaffold suggests that there may have been a single HGT event for GH32 followed by duplications over the course of evolution^31^. Our phylogenetic result for Biotype L suggests that the Hessian fly genome possibly gained MdesGH32 from its gut-associated microbial symbionts as a result of direct HGT or duplication from a single HGT event, during evolution. The ten MDL genes identified in the GP biotype were also acquired via HGT from bacterial symbionts^31^.

Virulent Biotype L larvae on susceptible host wheat exhibited dramatically high levels (> 400-fold) of *MdesGH32* mRNA compared to the avirulent larvae (< fourfold) on resistant wheat while a more modest increase (~ 12-fold) was observed in larvae feeding on Bd plants. Unlike host wheat, Bd plants are nonhost to Hessian fly^44^ and exhibit a physical and molecular response intermediate to resistant and susceptible host wheat^27,44^. The *MdesGH32* homolog in the Hessian fly biotype GP, *MDL-9*, had highest levels of transcripts, in the first- and second-instar larvae, in tissues involved in food digestion such as salivary glands and midgut^31^. Correlated with increased transcript levels, MdesGH32 protein was observed in the first-instar Biotype L larvae feeding on both the susceptible wheat lines used in this study. Interestingly, the larval MdesGH32 protein was detected in protein extracted from the feeding sites of both susceptible wheat lines (Newton and Centurk) after removing the larvae, indicating that MdesGH32 was secreted extra-orally by the larvae. Immunohistochemical analysis of the infested susceptible plant tissue revealed presence of MdesGH32 predominantly in the leaf sheath on which the larvae feed. Cross-sections of the tissue also revealed the presence of large regions of ruptured cells that form a nutritive tissue serving as a food source for the developing larvae. Presence of MdesGH32 around this region strongly suggests that it may also play a role in the hydrolysis of cell wall polymers in the plant to simpler nutritionally available sugar monomers, in addition to fructan digestion within the larval midgut as reported for MDL-9^31^. In fact, the same study revealed that levan and inulin levels are significantly higher at the feeding sites of Hessian fly larvae in infested susceptible wheat compared to uninfested wheat plants^31^. Indeed, enzyme activity assays that we carried out for the recombinant MdesGH32 revealed strong inulinase and invertase activities, that were also present in Biotype L larvae taken off susceptible wheat plants but not off resistant plants. It is quite possible that there are other PCWDEs that also function in the active breakdown of the cell walls. Several secreted salivary effector proteins from Hessian fly biotype GP larvae have been identified at the surface of feeding sites of the first-instar larvae on wheat plants using mass spectrometry, however, MdesGH32 was not one of them^32,38^, further corroborating the inter/intracellular location and activity of the insect-derived enzyme within the leaf sheath. Unlike the homolog MDL-9 from biotype GP^31^, MdesGH32 did not show any levanase activity, suggesting a modified role for the extra-oral MdesGH32 than MDL-9.

Coinciding with the temporal increase of the extra-oral MdesGH32 in the susceptible wheat crown tissue, the line Centurk (and Newton^26^) exhibited a striking increase in cell wall permeability over time following Hessian fly infestation compared to the resistant line Hamlet (and Iris^26^). The resistant plants exhibited only a transient permeability, limited in area. Induced permeability in the susceptible plants converts the entire crown tissue (larval feeding site), as evidenced by intense NR staining, into a nutrient sink that serves as a rich source of nutrients for the developing larvae^16,26^. Although results on cell permeability are correlated with increased MdesGH32 levels it is possible that there may be additional unidentified enzymes originating from Hessian fly^32,38^ or induced in the plant that may also be involved in the breakdown of the cell walls. In addition, there was an increase in glucose monomer levels in susceptible wheat plants by 5 and 8 DAH suggesting a breakdown of sucrose polymer into hexose monomers, at least partially, through the invertase activity of MdesGH32, enhancing the nutrient status of the larval diet and delivering a rich source of energy required by the developing larvae. The possibility that there may be additional or alternate currently unidentified factors that contribute to the increased glucose levels in susceptible wheat cannot be excluded. Interestingly, the increase in glucose monomers in the susceptible wheat plant also coincided with the increase in expression of MdesGLUT, a glucose transporter, in the virulent Biotype L larvae feeding on the susceptible wheat plant, suggesting that the rapid transport of plant nutrients across gut epithelial cells in the larvae from the lumen to the hemocoel is facilitated by MdesGLUT along with other transporter proteins and factors that regulate larval feeding. It is also interesting to note that fat bodies and eggs of Hessian fly contain increased levels of fructans, and the feeding stages show the highest levanase, inulinase and sucrase activities^31^, indicating that higher MdesGLUT activity may be required for transport of not only nutritionally acquired molecules but also endogenous simpler carbohydrates such as glucose and fructose. Glucose and related hexose transporters are the most abundant and common type of transporters in animals^45^. Other than a few isolated reports of a putative sucrose transporter in *Drosophila melanogaster*^46^, mouse^47^ and lobster^48^, sucrose transport has not been reported in animal cells. Additionally, in the Drosophila and mouse studies, the sucrose transport function was only demonstrated in *Saccharomyces cerevisae* with trehalose or the monosaccharides glucose, mannose and fructose as competitors for the sucrose uptake. It is likely that disaccharides in Hessian fly larvae are hydrolyzed to monosaccharides and transported across membranes via hexose transporters. Surprisingly, there was no corresponding increase in fructose monomer levels in the susceptible plants. However, we observed increased *TaFRK* expression in the susceptible plants that suggests phosphorylation of fructose to fructose 6-phosphate, which could then proceed into the glycolytic pathway and not be directly available to the larvae as a monosaccharide.

With significant cell wall rupture occurring, leading to the establishment of a nutritive tissue in susceptible wheat plants^16^, we examined the expression of 29 genes involved in the biosynthesis of cell wall polysaccharides and proteins in not only susceptible (Newton and Centurk) wheat lines but also two resistant (Iris and Hamlet) lines. In both susceptible wheat lines most genes were significantly down-regulated, suggesting that the virulent larvae block the susceptible plants from repairing the cell wall damage and contribute towards formation and maintenance of a nutrient sink. In resistant wheat lines, most of the genes, barring a few that showed increased transcript accumulation, were not differentially expressed, suggesting minimal cell wall damage is caused by the avirulent larvae and whatever little damage is caused does not spread.

**Figure 7:**
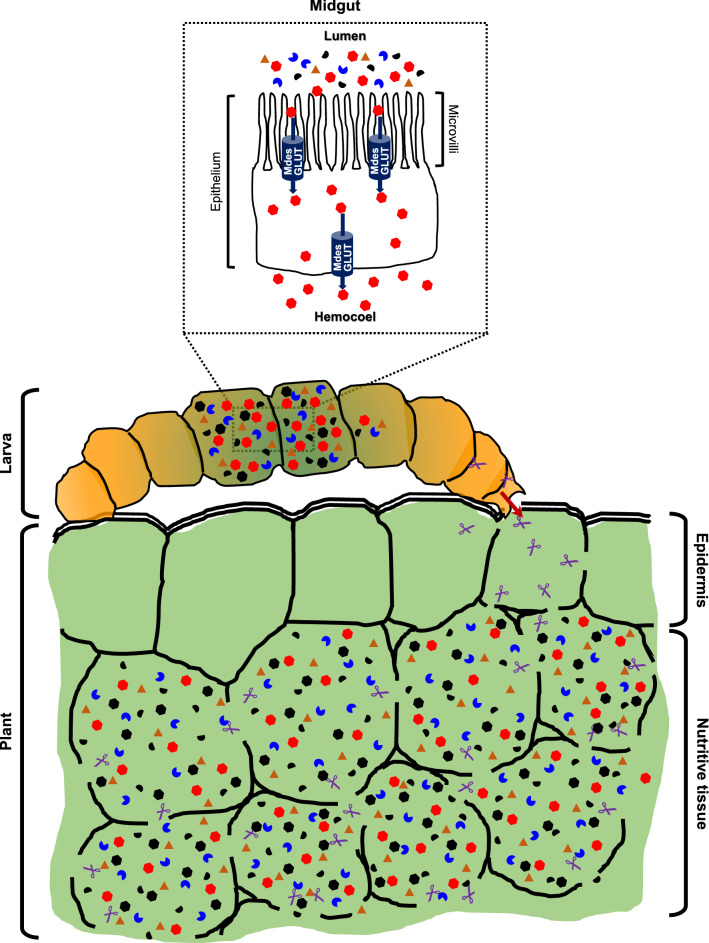
Model highlighting the role of MdesGH32 in the wheat-Hessian fly interaction. Virulent larva initiates feeding on the leaf sheath and secretes (red arrow) MdesGH32 (purple scissors) extra-orally into the plant tissue. MdesGH32 contributes towards the formation of a nutritive tissue by thinning and lysis of the epidermal and mesophyll cell walls by the inulinase activity against the plant cell wall inulin fructose polymers. Invertase activity of MdesGH32 converts sucrose, the main transport sugar into glucose (red filled hexagons) and fructose monomers that get phosphorylated (black filled hexagons). Rupture of the cell walls along with down-regulation of cell wall-associated genes increases permeability of the leaf tissue and creates a nutritive tissue rich in hexose sugar monomers, free amino acids (blue filled partial circles), polyamines (black filled semicircles) and miscellaneous solutes (orange filled triangles) that diffuse to the leaf surface where they are sucked up by the larva. Within the larval midgut (inset), the glucose transporter MdesGLUT (blue filled cylinder), actively transports plant- and insect-derived glucose from the lumen across the microvilli of the epithelial layer into the hemocoel.

Based on our results, we propose that the extra-oral secretion of MdesGH32 into the susceptible host plant also facilitates the breakdown of inulin and other fructan polymers into monomers thereby playing an important role in establishing susceptibility and a nutrient sink in wheat plants. Hessian fly larvae have minuscule mandibles that are used for puncturing the plant cell wall^49^ but cannot be utilized for chewing^50^. Therefore, it is crucial that nutrition is available to the developing larvae in a liquid form rather than solid form. The formation of a nutritive tissue^16^ is one of the primary steps in this process. Thinning and lysis of the epidermal cell walls^16^ in the leaf sheath being probed by the larvae may be accomplished by the cumulative action of the extra-oral secretion of MdesGH32 from the larvae into the leaf sheath via inulinase activity against the plant cell wall inulin fructose polymers, and other, currently unidentified, PCWDEs, causing the walls to rupture. In addition, the invertase activity of MdesGH32, along with possibly other Hessian fly- or plant-derived enzymes, may be responsible for the conversion of the primary transport sugar in plants, sucrose, into glucose and fructose monomers adding to the nutritional quality of the energy source (Fig. 7). The increased permeability as detected by NR staining in susceptible wheat and the suppression of cell wall-associated genes, further corroborates the breakdown of the plant cell wall that is unable to repair itself, while increased levels of glucose monomers in susceptible wheat provide evidence of a readily available energy source for the larvae. In fact, in addition to carbohydrates (this study and Cheng et al.^31^), susceptible wheat plants are a rich source of free amino acids as well as polyamines^15,17,18^ that are available to the virulent larvae. The permeability caused by the rupture of the cell walls of the nutritive tissue may allow the nutrients to diffuse to the leaf surface where the virulent larvae are able to suck these up, thus establishing susceptibility.

## Methods

### Insect and plant material

The Hessian fly (*Mayetiola destructor*) Biotype L stock was obtained from the USDA-ARS Crop Production and Pest Control Research Unit in West Lafayette, IN^51^. Wheat (*Triticum aestivum*) lines ‘Iris’ (harboring *H9* resistance gene), ‘Hamlet’ (harboring *H21* resistance gene), ‘Newton’ and ‘Centurk’ (containing no *H* resistance genes) were used in the study. Biotype L Hessian fly larvae on wheat lines, Iris and Hamlet, yield an incompatible (resistant wheat, avirulent larvae) interaction and on Newton and Centurk yield a compatible (susceptible wheat, virulent larvae) interaction. *Brachypodium distachyon* (Bd) seeds of line Bd21show nonhost resistance to Biotype L infestation^27^.

### Plant growth and infestation

Twelve seeds (per pot) for each wheat line were planted in 4-inch pots containing Promix Professional growing medium (Premier Horticulture Inc., Quakertown, PA, USA). Plants were grown in a Conviron growth chamber (Controlled Environments Limited, Winnipeg, Manitoba, Canada) as described in Subramanyam et al.^18^. Plants at the 1-leaf stage were infested with 3 female and 2 male Hessian flies. For Bd plants, 10 seeds were planted in each 4-inch pot containing equal volume (50:50 mix) of growing medium: vermiculite (Perlite Vermiculite Packaging Industries, North Bloomfield, OH) and Farfard professional potting mix (Conrad Farfard Inc., Agawam, MA) and grown in a Conviron growth chamber as previously described^44^. A few uninfested pots of each wheat line and Bd plants were kept as controls.

### Insect and plant tissue collections

Biotype L larvae feeding on Iris and Newton wheat lines, and Bd plants, were collected. Neonate (newly hatched and non-feeding) larvae were collected in triplicate from 30 plants per replicate. For collections of avirulent Hessian fly larvae, first instar Biotype L larvae feeding on Iris (resistant) wheat were collected only on 1 and 3 days after egg hatch (DAH) as the avirulent larvae die by 4–5 DAH. For virulent larval collections, first- (1, 3 DAH) and second- (9 DAH) instar Biotype L larvae feeding on Newton (susceptible) wheat plants were collected in triplicates from 30 plants per time point per replicate. Biotype L larval collections feeding on Bd plants included the neonates, first- (3 DAH) and second-instars (9 DAH) in three biologically replicated experiments per time point.

For plant tissue collections from wheat lines, the 1st leaf was carefully discarded and the crown tissue was harvested from leaf 2 from Hessian fly-infested and uninfested plants at selected time points (DAH). For gene expression studies, tissue was collected from three biological replicates with at least 20 plants per time point per replicate. The time points for collections varied for each experiment. The tissues were frozen in liquid nitrogen and stored at − 80 °C.

### Quantitative real-time reverse transcription PCR analyses

Larval and plant RNA was isolated using TRIzol reagent (Invitrogen, Carlsbad, CA) as per manufacturer’s protocol. The RNA preps were subjected to DNase-treatment using TURBO DNA-free kit (Invitrogen). cDNA for transcript quantification was generated by reverse transcription using random hexamers for Hessian fly larval samples^15^ and oligo dT for plant samples^28^. Transcript profiling was undertaken using quantitative real-time reverse transcription PCR (qRT-PCR). Primers for qRT-PCR were designed from gene sequences with Primer Express 3.0 software from Applied Biosystems (ABI, Foster City, CA). The qRT-PCR was performed on a LightCycler 480 Instrument II (Roche Diagnostics Corporation, Indianapolis, IN) as described in Hargarten et al.^44^. Reaction volume of 10 µl contained 5 µl of 2X SensiFAST SYBR No-ROX mix (Bioline, Taunton, MA), gene-specific forward and reverse primers at a final concentration 0.4 µM each and 20 ng cDNA template. The PCR cycling parameters were 40 cycles of 95 °C for 5 s, 60 °C for 10 s, and 72 °C for 20 s. Amplification of a single product was confirmed through melt curve analysis (95 °C for 5 s, 65 °C for 1 min, 97 °C continuous) following PCR. qRT-PCRs were performed in triplicate for all three biological replicates. Hessian fly 18S rRNA and wheat Ubiquitin genes were included in qRT-PCR as the endogenous controls for larval and wheat samples, respectively along with no-template negative controls. Relative Standard Curve method (ABI User Bulletin 2, ABI PRISM 7700 Sequence Detection System) was used for quantification of transcript abundance. One-sided Tukey pairwise comparison (JMP Pro Ver. 14, SAS Institute Inc.) was used to determine significant differences in relative expression values. Differences were considered statistically significant at *p* < 0.05. Fold change of expression in Hessian fly larval samples was calculated as the ratio of transcript levels in larvae that have started feeding after egg hatch to the neonates that are assumed to be non-feeding. Fold change in expression in wheat tissue was calculated as the ratio of transcript levels in infested samples to the uninfested controls at the respective time points.

### Quantification of *MdesGH32* in larval tissue

*MdesGH32* transcripts were quantified from Biotype L Hessian fly larval samples feeding on wheat and Bd plants. The qRT-PCR forward (5′-TTGAAATGACGGTCGACCTAAA-3′) and reverse (5′-GAACCAGAAAACACAAAATTGCAT-3′) primers were designed from transcript sequence of Mdes007867, mapped to A1 chromosome on the annotated Hessian fly (GP Biotype) genome^32^ and encoding a GH32 family protein. The primer sequences were specific for *MdesGH32* and did not show significant similarity to any of the other MDL gene homologs^31^. The transcripts were quantified in Biotype L larvae feeding on (i) Iris wheat, 1 and 3 DAH; (ii) Newton wheat, 1, 3 and 9 DAH; and (iii) Bd plants, 3 and 9 DAH. The transcripts were also quantified in the neonates on wheat and Bd plants. For qRT-PCR with Hessian fly, 18S ribosomal RNA gene was used as the endogenous control and forward (5′ CACGCGCGCTACAATGAA-3′) and reverse (5′-ACGGTTTACCCGAGCCTTTAG-3′) primers were designed from GenBank accession number KC177284.

### Quantification of *MdesGLUT* in larval tissue

*MdesGLUT* transcripts were quantified in avirulent (first-instar) and virulent (first- and second-instar) Biotype L Hessian fly larval samples feeding on resistant and susceptible wheat, respectively. The qRT-PCR forward (5′-TGATTGGGCTTCGAAATTGTT-3′) and reverse (5′-AACGCATAAAACCATGTAAATGATACC-3′) primers were designed from the transcript sequence of Mdes018438, annotated from Hessian fly (biotype GP) genome^32^ and encoding a putative glucose transporter (GLUT) protein. Transcripts were also quantified in the neonates on wheat plants and Hessian fly 18S ribosomal RNA was used as an endogenous control gene as described previously.

### Quantification of *TaFRK* and cell wall-associated genes in wheat tissue

To understand the rapid turnover of fructose and determine the expression of genes encoding proteins involved in biosynthesis of cellulose, hemicellulose and pectin, qRT-PCR was employed as described above for *TaFRK* encoding a putative fructokinase, and 29 cell wall-associated genes. Both studies were undertaken in two resistant wheat lines, Iris and Hamlet, and their susceptible counterparts, Newton and Centurk, respectively. The expression of *TaFRK* was quantified at 1, 3, 5 and 8 DAH time points in infested and uninfested wheat lines, while an additional time point of 0.5 DAH was studied for expression of the cell wall-associated genes. Primers (Supplementary Table 2) to carry out qRT-PCR for the cell wall-associated genes were designed from sequences for the gene IDs obtained from the annotated *Triticum aestivum* genome sequence^52^, while *TaFRK* primers were designed from Affymetrix probe id Ta.9577.1.S1_at (GenBank accession no. XM_020293052.1). Wheat ubiquitin (GenBank accession no. X56803) gene was used as the endogenous control. Expression data for cell wall-associated genes are represented as a heatmap generated using R in Bioconductor (https://www.bioconductor.org/).

### Isolation of cDNA and genomic clones of *MdesGH32*

The transcript sequence of Mdes007867 was used as the starting point to clone the coding and genomic sequences of *MdesGH32*. The JBrowse predicted start and stop codons from Mdes007867 sequence were used to design the forward (5′- ATGTTAAAAATCAATATTTTAATCGTTGCATTTTC-3′) and reverse (5′- TCACTCGCTACACGGAAACTTATC-3′) primers for PCR amplification. The cDNA synthesized from total RNA (described above) from virulent Biotype L larvae (3 DAH time point) feeding on Newton wheat line was used as the template for PCR amplification of *MdesGH32* coding sequence. Genomic DNA was extracted from Biotype L larvae (3 DAH time point) feeding on Newton wheat line using DNeasy Blood & Tissue Kit (Qiagen, Valencia, CA) and used as template for PCR to obtain the genomic clone of *MdesGH32*. A 25 µl reaction mixture was set up containing 1X PCR buffer, 2 mM MgSO_4_, 0.2 mM of each dNTP, 0.2 µM of each primer, 50 ng cDNA template, and 1 unit of Platinum *Taq* DNA Polymerase High Fidelity (Invitrogen, Carlsbad, CA). The PCR cycling parameters were as follows: 94 °C for 2 min; 35 cycles of 94 °C for 30 s, 50 °C for 30 s, 68 °C for 2 min (cDNA) or 5 min (genomic DNA); final extension of 68 °C for 5 min. QIAquick Gel Extraction Kit (Qiagen) and TOPO-TA Cloning Kit for Sequencing (Invitrogen) were used to purify and clone each amplicon, respectively. Cloned products were sequenced by the Purdue University Genomics Core Facility. The coding and genomic sequences (GenBank accession no. MT179729) were submitted to the National Center for Biotechnology Information (NCBI). NCBI BLAST programs (http://www.ncbi.nlm.nih.gov/) were used for annotation and sequence similarity analyses of *MdesGH32*. The *MdesGH32* ORF was used to predict the isoelectric point (pI) and molecular mass utilizing the pI/MW tool at Expasy (www.expasy.org/tools.pi_tool.html). The NCBI blastp tools were used for conserved domain searches. Signal peptides were predicted using SignalP v3.0 (Center for Biological Sequence Analysis, Technical University of Denmark; http://www.cbs.dtu.dk/services/SignalP/). The 3D structure of MdesGH32 was predicted using Phyre2 (Protein Homology/Analogy Recognition Engine V2.0)^53^.

### Phylogenetic analysis of MdesGH32

To determine the evolutionary relationship of MdesGH32, the deduced protein sequence was used to query the NCBI non-redundant protein library (Supplementary Table 3). The blastp search^54^ was carried out against Bacteria (taxid:2), Fungi (taxid:4751), Dicotyledoneae (taxid:71240), Monocotyledoneae (taxid:4447), and Insecta (taxid:50557) containing members of Coleoptera (taxid:7041), Lepidoptera (taxid:7088), and Diptera (taxid:7147). The top 10–12 hits in each category with query coverage of 90% and above were included in the phylogenetic analysis (Supplementary Table 3). All sequences were aligned using MUSCLE (Multiple Sequence Comparison by Log-Expectation) 3.8.31^55^, and maximum-likelihood phylogeny estimated using PhyML 3.0 based on an Approximate Likelihood-Ratio Test^56^. The phylogenetic tree was drawn using the tree viewer, TreeDyn 198.3^57^.

### Purification of recombinant MdesGH32 protein

Recombinant expression plasmid was constructed by directionally cloning the ORF (excluding the 19-amino acid signal peptide sequence) of MdesGH32 into pET-30a (+) expression vector (GenScript, Piscataway, NJ), in frame with a 6 × N-terminal Histidine tag. *Escherichia coli* BL21 Star (DE3) competent cells were transformed with the recombinant expression plasmid of MdesGH32. A single colony was inoculated in Luria–Bertani medium at 37 °C containing 50 µg/mL kanamycin, and isopropyl-*β*-d-thiogalactopyranoside (0.5 mM) was added to induce protein expression. The recombinant protein was purified from the supernatant lysate using one-step purification by Ni^2+^ NTA resin. Eluted MdesGH32 recombinant protein was stored in Phosphate Buffer Saline (pH 7.4). All purification procedures were carried out at 4 °C. Protein concentration was determined by Bradford Assay (ThermoFisher Scientific, Waltham, MA).

### Extraction of total protein

Total protein was extracted from Hessian fly larvae and host plant samples. For plant samples, seedlings of susceptible wheat lines Newton and Centurk were grown and infested at 1-leaf stage with Biotype L Hessian flies as described above. The 1st leaf was carefully discarded and the crown tissue was harvested from leaf 2 at 3, 5 and 8 DAH from 5 plants per time point. The harvested crown tissue was immersed in deionized water and gently agitated to wash off the larvae. The tissues were frozen in liquid nitrogen and stored at − 80 °C. The Plant Protein Extraction Kit (ThermoFisher Scientific) was used to extract total plant protein from 200 mg of frozen ground tissue. For Hessian fly samples, Biotype L larvae were removed from resistant wheat (Iris) plants at 3 DAH and from two susceptible wheat (Newton and Centurk) lines at 3, 5 and 8 DAH. Total larval protein extraction was done using the Minute Total Protein Extraction Kit (Invent Biotechnologies Inc., Plymouth, MN). Protein estimation for the plant and insect samples was done using the MicroBCA Protein Assay kit (ThermoFisher Scientific).

### Protein gel blot and immunodetection of MdesGH32

Affinity-purified polyclonal antibodies against MdesGH32 protein were produced commercially (GenScript) against an 18-amino acid custom-synthesized peptide (LYDEKYRPQIHFSPPNGW; Fig. 1b) in New Zealand white rabbits as per standardized procedures. The peptide sequence was specific for MdesGH32 and did not share more than 66.7–83.3% identity with other MDL gene homologs. Total larval, plant, and recombinant proteins were resolved by SDS-PAGE, using 4–20% Mini-PROTEAN TGX Precast Gel (Bio-Rad, Hercules, CA) as per manufacturer’s instructions. Precision Plus Kaleidoscope (Bio-Rad) protein standard was used as a molecular weight ladder. Protein gel blot and immunodetection of MdesGH32 was performed as per Subramanyam et al.^22^. The membranes were probed with anti-MdesGH32 as a primary antibody or the pre-immune serum (as a negative control) at a dilution of 1:1000 and goat anti-rabbit horseradish peroxidase (HRP)-conjugated IgG (ThermoFisher Scientific) as the secondary antibody at a dilution of 1:10,000. Protein signal was detected using SuperSignal West Pico Chemiluminescent substrate (ThermoFisher Scientific), and luminescence was exposed to BioMax MR film (ThermoFisher Scientific).

### Immunohistochemical detection of MdesGH32

The extra-oral secretion of MdesGH32 within wheat plant tissue was detected by immunohistochemical staining. Crown tissue (feeding site) was harvested from Biotype L infested and uninfested Newton (susceptible) wheat plants at 7 DAH as described previously. Harvested tissues were fixed and embedded in Paraplast (Sigma-Aldrich, St. Louis, MO) using the method described^58^. The tissue was sectioned at a thickness of 4 µm using a HM 355S microtome (ThermoFisher Scientific). The sections were mounted on charged slides and dried at 60 °C. The antigen retrieval and chromogen staining with 3,3′-Diaminobenzidine (DAB) was performed as described^59^. The primary antibody (anti-MdesGH32, or the pre-immune serum as negative control) and Rabbit IgG isotype control (Vector Labs, Burlingame, CA) were used at a dilution of 1:100 and 1:5000, respectively. Immunostaining was done using ImmPRESS HRP Goat Anti-Rabbit IgG Polymer Detection kit, Peroxidase (Vector Labs). The slides were counterstained with hematoxylin for better contrast to the chromogen. Images were taken using Versa8 whole-slide scanner (Leica Biosystems, Buffalo Grove, IL).

### Monosaccharide levels in Hessian fly-infested wheat seedlings

Monosaccharides glucose and fructose were commercially quantified in Hamlet (resistant) and Centurk (susceptible) wheat seedlings using ultrahigh performance liquid chromatography-mass spectrometry (UPLC-MS) by Creative Proteomics (Shirley, NY). The plants were grown and infested with Biotype L, and crown tissue harvested as described above. A few plants of Hamlet and Centurk were left as uninfested controls. Crown tissues were harvested from 20 infested and uninfested control plants at 5 and 8 DAH, frozen in liquid nitrogen, and crushed to a fine powder using mortar and pestle. To extract the sugars, 100 mg of crushed crown tissue was transferred into an Eppendorf tube along with 75% methanol (10 µl/mg). After addition of two 5 mm stainless steel balls the tissue was homogenized on a Retsch MM400 Mixer Mill (Haan, Germany) followed by sonication in a water bath for 5 min. The samples were clarified by centrifugation at 21,000×*g* at 10 °C for 15 min. Standard and test solutions were mixed with internal standards containing 6 U-^13^C-labeled analogues for glucose and fructose as per manufacturer’s protocol (Creative Proteomics). The mixtures were allowed to react at 50 °C for 60 min. Each reactant was mixed with 1.8 mL of 20% methanol and 5 µl injected into a UPLC-(-) ESI-MRM/MS (Agilent 1290 UHPLC system coupled to an Agilent 6495 QQQ mass spectrometer) system using a multiple-reaction monitoring (MRM) mode with negative-ion detection. Analyte-to-internal standard peak area ratios (As/Ai) versus molar concentrations (nmol/mL) of standard solutions for each sugar were used to construct linear calibration curves. The concentration of individual sugars was calculated with internal calibration from their standard calibration curves with the As/Ai values measured for each sample. The data were collected from three biological replicates for each sample and statistical analysis was carried out using the one-sided Tukey pairwise comparison (JMP Pro Ver. 14, SAS Institute Inc.) for sugar levels between infested and uninfested samples at respective time points.

### Enzyme activity assays

Invertase, inulinase and levanase activities were assayed in total larval protein extracted from Biotype L larvae feeding on Iris at 3 DAH, Newton and Centurk at 3, 5 and 8 DAH, and the MdesGH32 recombinant protein, using glucose standard. Enzyme activity in the larval samples would be the cumulative activity of all invertases or inulinases or levanases in the larvae. Invertase activity assay was carried out using Invertase Activity Colorimetric Assay (BioVision Inc., Milpitas, CA) as per manufacturer’s instructions. One unit of invertase activity was defined as the amount of enzyme that generates 1 µmol of glucose min^−1^ at pH 4.5 and 37 °C. Inulinase and levanase activities were assayed by incubating samples with 1% (w/v) inulin (Megazyme Biotechnology, Bray, Ireland) and 1% (w/v) levan (Megazyme) substrates, respectively, in 0.05 M sodium acetate buffer at pH 5. Dinitrosalicylic acid method (Miller, 1959) was used to detect reducing sugars released during incubation. The absorbance was measured at 540 nm using the Multiskan Sky Microplate Spectrophotometer (ThermoFisher Scientific). Levanase and inulinase assays were carried out at 50 °C and 40 °C, respectively, for 30 min. One unit of levanase and inulinase activity was defined as the amount of enzyme that generates 1 µmol of glucose min^−1^ at optimal pH and temperature.

### Neutral red staining

Neutral red staining was carried out as described in Williams et al.^26^ and Subramanyam et al.^27^. Hamlet (resistant) and Centurk (susceptible) wheat lines were grown and infested with Biotype L Hessian fly as described above. At 3, 5 and 8 DAH, 10 infested plants from each line were cut below the root/crown junction, and 1st leaf gently peeled back exposing leaf 2. Dissection of the plants was done carefully to avoid wounding. Staining was carried out for 10 min in 0.1% NR (Sigma-Aldrich), followed by thorough rinsing with water. Uninfested plants were pierced with a 0.2 mm minuten pin before staining as positive controls. Intensity of staining was scored as per Williams et al.^26^, with “0” indicating no stain and “7” indicating a completely red crown. Results were captured with a DP27 camera system on SZX2 stereomicroscope (Olympus America Inc., Center Valley, PA).

## Supplementary Information

Supplementary Information 1.

Supplementary Table 3.

## Data Availability

The authors declare that all data supporting the findings of this study are available within the paper and any raw data can be obtained from the corresponding author on request.
