# Supplementary material for: Insect derived extra oral GH32 plays a role in susceptibility of wheat to Hessian fly
Source: Sci Rep. 2021 Jan 22;11:2081. doi: 10.1038/s41598-021-81481-4 (PMC7822839; doi:10.1038/s41598-021-81481-4)
Supplement: Supplementary file 1 — Supplementary Information 1. [file 41598_2021_81481_MOESM1_ESM.pdf]

Insect derived extra oral GH32 plays a role in susceptibility of wheat to Hessian fly

Subhashree Subramanyam[1,2*], Jill A. Nemacheck[1,2], Victor Bernal-Crespo[3], Nagesh Sardesai[4]

[1]Crop Production and Pest Control Research Unit, USDA-ARS, West Lafayette, IN, USA

[2]Department of Entomology, Purdue University, West Lafayette, IN, USA

[3]College of Veterinary Medicine, Purdue University, West Lafayette, IN, USA

[4]Corteva Agriscience, Johnston, IA, USA

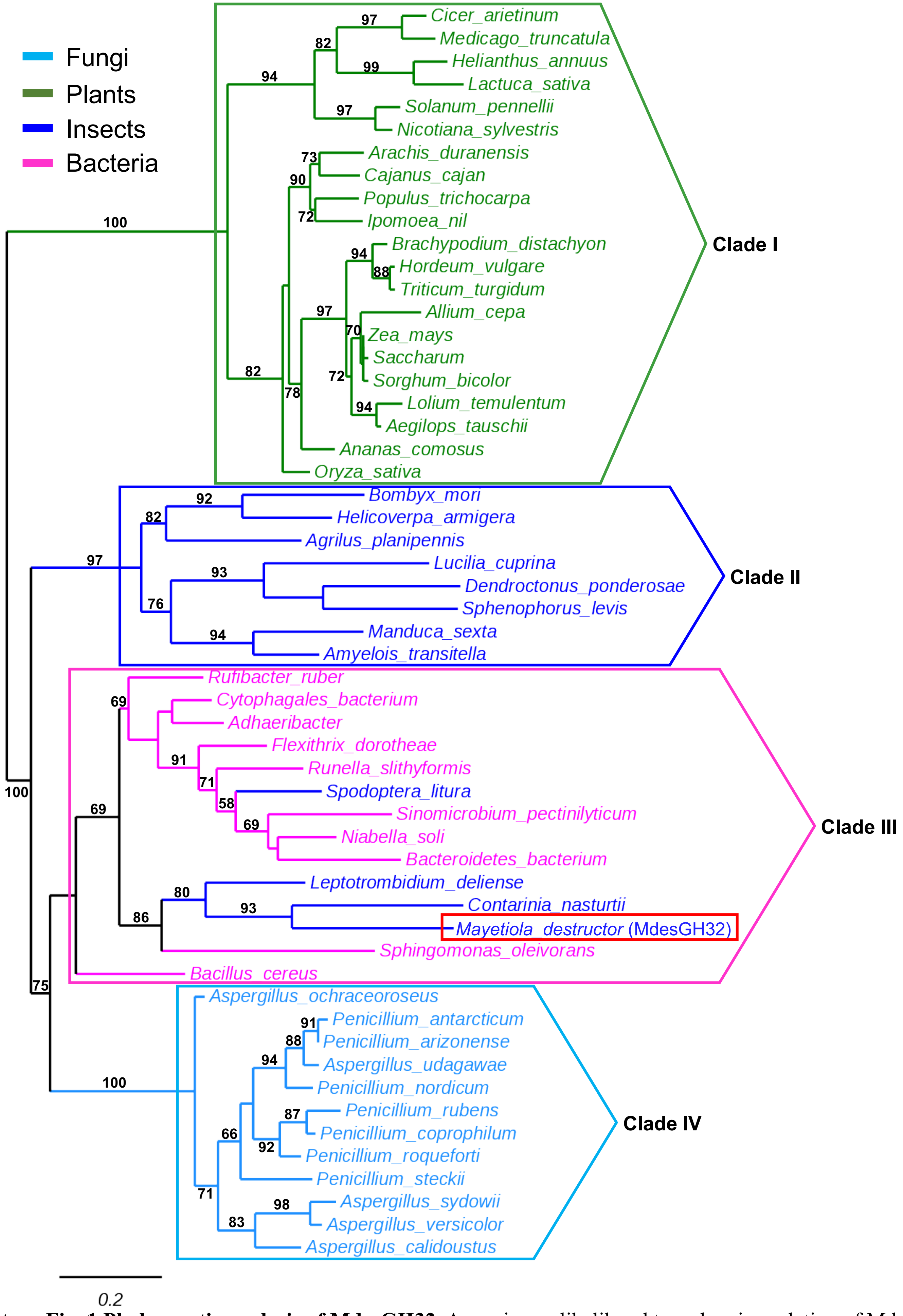

Supplementary Fig. 1 Phylogenetic analysis of MdesGH32. A maximum likelihood tree showing relation of MdesGH32 (red box) with orthologs of GH32s derived from other plants (green), insects (dark blue), bacteria (purple) and fungi (light blue). The NCBI accession numbers for these sequences are provided in Supplementary Table 3. Bootstrap values (1000 replicates) greater than 50% are shown next to the branches. Scale bar represents branch lengths.

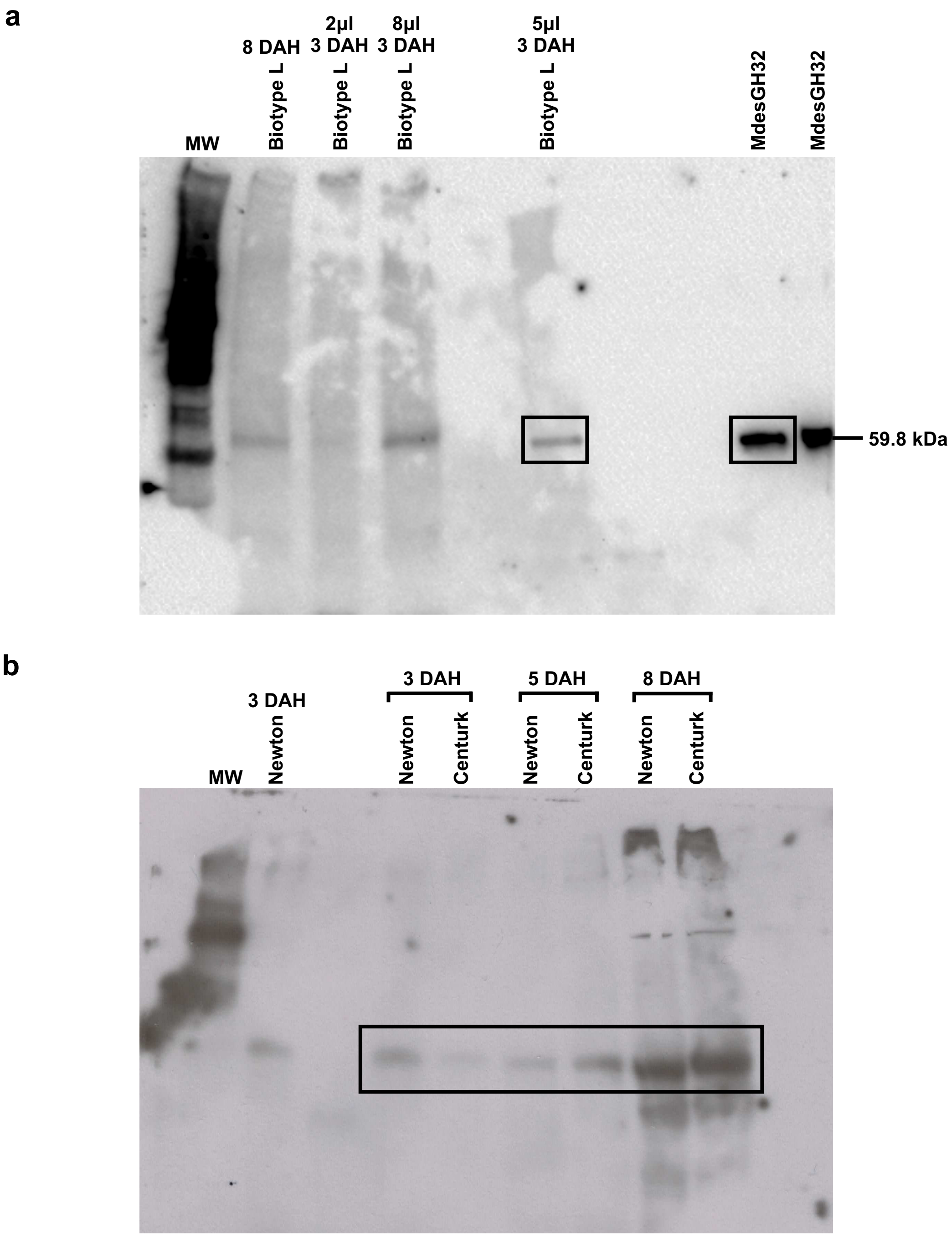

Supplementary Fig. 2 *MdesGH32* expression in Hessian fly larvae and wheat plants. a, Gel blot showing the immunodetection of MdesGH32 in virulent Biotype L larvae, 3 and 8 DAH. Different volumes (2, 5 and 8 µl) of the protein were loaded on the gel for the 3 DAH sample. Purified recombinant MdesGH32 protein was used as a positive control (last two lanes) and showed a single band of the expected size of 59.8 kDa. **b**, Gel blot showing the immunodetection of MdesGH32 in susceptible wheat plants (Newton and Centurk) 3, 5 and 8 DAH. Black boxes indicate the regions of the gel blots taken to form the composite image in Figure 2d. MW, molecular weight ladder.

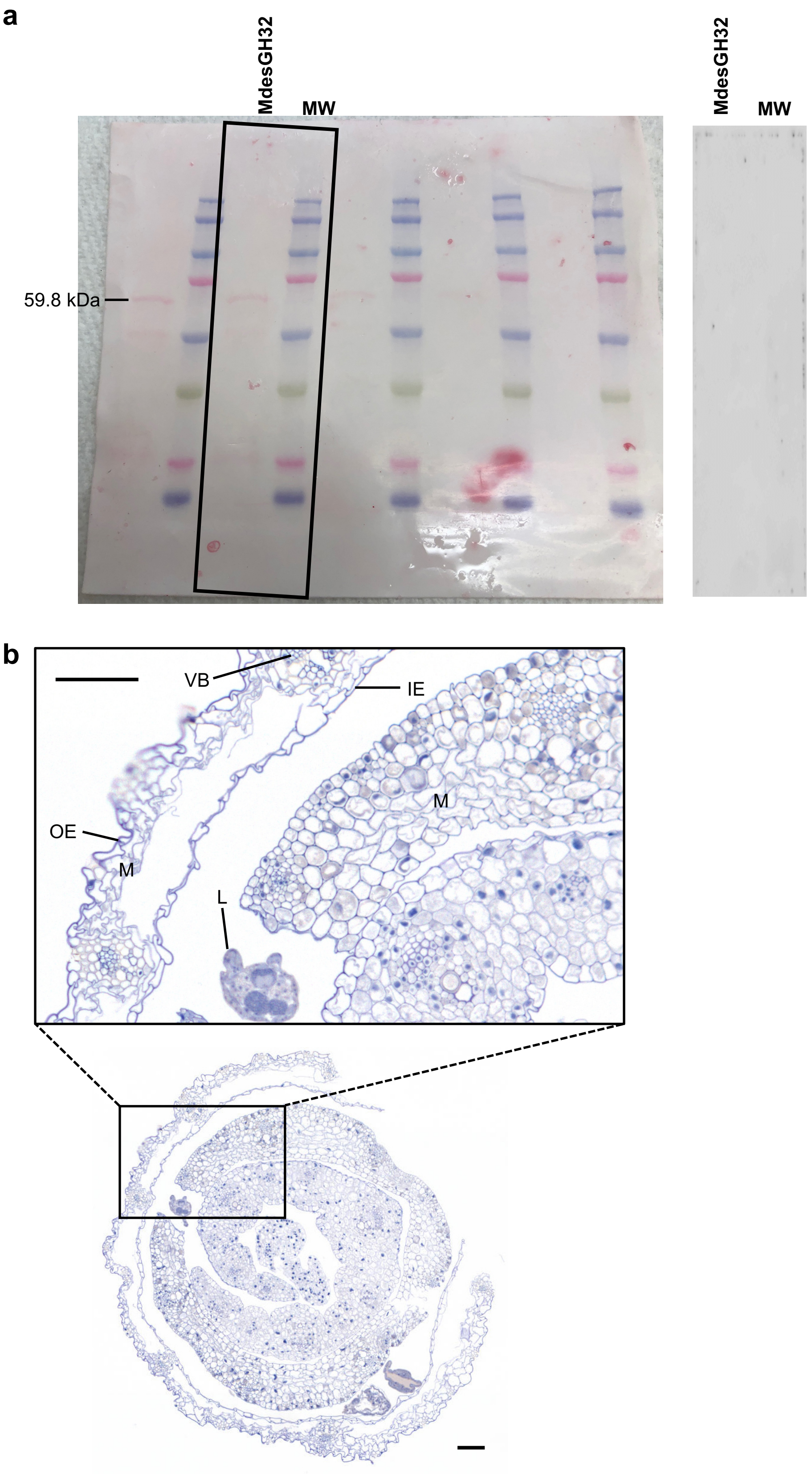

Supplementary Fig. 3 Preimmune MdesGH32 serum specificity. a, Gel-blot of recombinant MdesGH32 and molecular weight (MW) ladder stained with Ponceau (left panel) and probed with preimmune serum for MdesGH32 antibody (right panel) showing no reactivity. A strip of the Ponceau-stained blot shown in the black box was cut out and probed with preimmune serum for MdesGH32 antibody. **b**, Immunohistochemical localization of preimmune MdesGH32 serum in Biotype L-infested Newton crown tissue collected 8 DAH showing lack of non-specific staining throughout the mesophyll cells (M) between the outer epidermis (OE) and inner epidermis (IE) of the leaf sheath (inset) being fed on by larvae (L). VB, vascular bundle; scale bar, 100 µm.

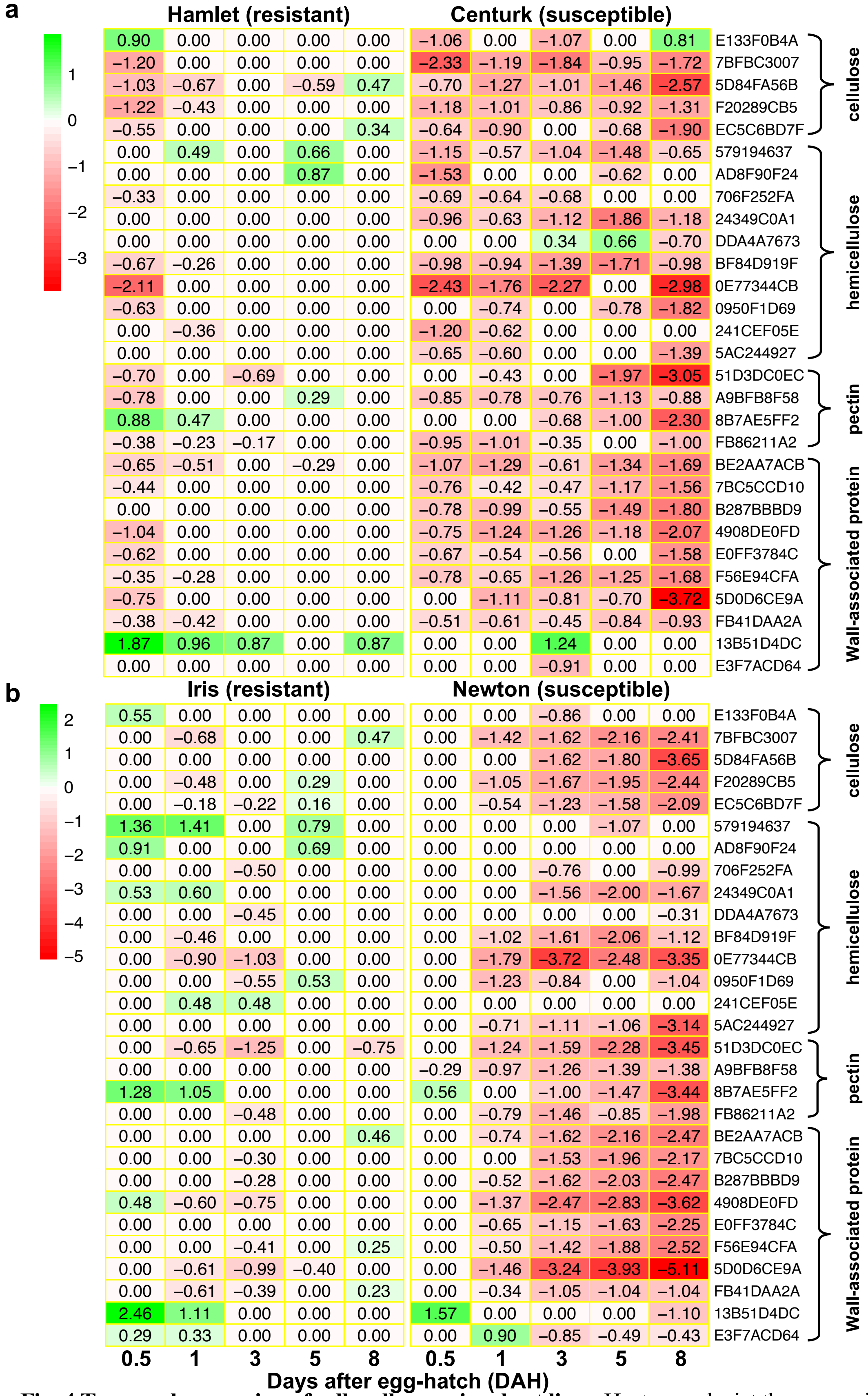

Supplementary Fig. 4 Temporal expression of cell wall genes in wheat lines. Heatmaps depict the expression profile of 29 genes encoding proteins, grouped by function, involved in the biosynthesis of cell wall in **a**, Hamlet and Centurk; **b**, Iris and Newton. Gene IDs are listed with the prefix "Traes" removed for brevity. Green and red represent up-regulated and down-regulated genes, respectively, while genes not differentially expressed are indicated in white. Logarithmic fold change values are given in each cell of the heatmap. Data are from three biological replicates (*n*=3; each with three technical replicates).

Supplementary Table 1. Neutral Red scoring* of Hessian fly-infested wheat plants

DAH	Resistant Wheat (Hamlet)	Susceptible Wheat (Centurk)
3	3.1± 0.8	4.4 ± 1.8
5	3.3 ± 0.5	6.1 ± 1.3
8	3.2 ± 0.4	6.5 ± 1.0

* Intensity of Neutral Red stain was scored on a scale of 0-7, according Williams et al[26].
DAH: Days after egg hatch

Supplementary Table 2. qRT-PCR primers for wheat genes

Gene ID	Annotation	Forward (5'-3')	Reverse (5'-3')
**Endogenous control**			
TaUBQ	ubiquitin	ggtgtctccggtatcctccaa	tgctccacaccagcagaag
**Fructose metabolism**			
TaFRK	fructokinase	caaggacgactccatcttctacaat	ccgcaagcgttcgagaac
**Cell wall-associated**			
**Cellulose**			
Traes_5DL_E133F0B4A	cellulose synthase	ggaaaaaccaagctacgacatca	agtgagttgggtgcccatagtc
Traes_2DS_7BFBC3007	cellulose synthase	cgtcgggctcgtgtatgtc	gaagctgcaacttctgaaaaatca
Traes_7AS_5D84FA56B	sucrose synthase	ggcgtgcagttcctcaaca	gggtacatgctctccttgtcatg
Traes_5AS_F20289CB5	COBRA	aagacgcaggcgctgatg	gctgttgtagaaggaggagaagga
Traes_2DS_EC5C6BD7F	COBRA	ccgaggtcttcagcttcgacta	gcgccgtatcattgatgga
**Hemicellulose**			
Traes_1AL_579194637	expansin	caaagggtgccatgcaatc	ccgctcgacgtggaagtt
Traes_7DL_AD8F90F24	xyloglucan endotransglucosylase	ggcgcgtggatgtacca	cgcagtagttgtagatcatgtagttcttc
Traes_6AS_706F252FA	endoglucanase	atccgcccctggaacaa	gtggacggtgaggaggaaga
Traes_2AL_24349C0A1	beta-glucosidase	acggcgtgacaattggaaa	accttccggaacatcatagtaac
Traes_3B_DDA4A7673	alpha-glucosidase	gcgtgaggacgatgacatactg	cctcgcgttccccttctc
Traes_1AL_BF84D919F	beta-galactosidase	ctgaaacctatagacaacctccttgtt	cgttatctccagcggattgc
Traes_6DL_0E77344CB	alpha beta-mannosidase	gaagcagccctgcaggaa	tgttagccttatttgtgagcaatctt
Traes_4DL_0950F1D69	xylosidase	tcaaggccgggttggat	catgccgtgcacctgtgt
Traes_7AS_241CEF05E	glycosyltransferase	ccccagcggagacagaaa	gccaccatctggcttggt
Traes_4BS_5AC244927	xylanase	gcgccaggtgtccaacag	gaacacggcccagttgaagt
**Pectin**			
Traes_5BL_51D3DC0EC	pectin lyase	ttgccttagagaacatcgacctt	ctggcaagtactctcggtgtca
Traes_5BL_A9BFB8F58	pectin acetylesterase	acgcatcccagatgaaagtctt	ccttgcacaattcccaccat
Traes_6DL_8B7AE5FF2	pectin esterase	ccgaggccagcaacttca	ggtcggcagccacatgtt
Traes_4DL_FB86211A2	pectin esterase	tggcaccgggtggatatg	cgaatacaacacgggagtaaggt
**Wall-associated protein**			
Traes_7AS_BE2AA7ACB	rhamnogalacturonate lyase protein	tgatgtgatcaaaggcacagagt	aaggagacctcagcctggtttt
Traes_7AS_7BC5CCD10	rhamnogalacturonate lyase protein	ctgtgcctgccgctaactc	atacggctaaggcaggctgat
Traes_7DS_B287BBBD9	rhamnogalacturonate lyase protein	ctatctgaagcagcccaggaa	ggcacagcacagggtcctt
Traes_1DS_4908DE0FD	arabinogalactan protein	agtccggcaccctcaactc	ctgggagaccacgtggaact
Traes_5BL_E0FF3784C	arabinogalactan protein	cgccgtctactccatcaaca	gcgggtccttgctgaaga
Traes_4DL_F56E94CFA	hydroxyproline-rich protein	ccgctttgccttccgaata	ttccggcaaccttcatcaa
Traes_2DS_5D0D6CE9A	proline-rich protein	tccgcgtggcgatca	accgccttgctctcgtactc
Traes_6AL_FB41DAA2A	wall-associated kinase	tgcaagctcaccgacaagag	aggaactgcgacgagaggtact
Traes_6DL_13B51D4DC	wall-associated kinase	tgcagaagcggaagctcat	gcctccgttgtgctcgaa
Traes_7DS_E3F7ACD64	polygalaturonase inhibitor	gctctcctggccgtcaag	gcgcggtggagttcca
